# The Mind-Evolution Problem: The Difficulty of Fitting Consciousness in an Evolutionary Framework

**DOI:** 10.3389/fpsyg.2018.01537

**Published:** 2018-08-24

**Authors:** Yoram Gutfreund

**Affiliations:** Department of Neurobiology, The Rappaport Research Institute and Faculty of Medicine, Technion – Israel Institute of Technology, Haifa, Israel

**Keywords:** sentience, evolution, natural selection, cognitive ethology, consciousness, animals (human and non-human)

## Introduction

Evolution gave rise to immensely complex and diverse embodied biological systems called animals, which behave adaptively to survive and produce. At least one out of millions of species on the planet has a remarkable and mysterious capability not only to behave but also to sometimes feel that it is behaving^1^. We know this because we belong to this species, and as noted, nothing is more real than one's own feelings (Chalmers, 1996). This capability is called phenomenal consciousness, conscious awareness, or sentiency (we will refer to it here as consciousness). Scientific agreement is that consciousness arises from the brain's activity, however, there is no understanding as to how (Revonsuo and Kamppinen, 2013). Intuitively, there needs to be a property in our brain that gives us this capability, a property that has evolved. Identifying this property and tracking its evolution is the key to understanding the evolution of consciousness.

If consciousness is indeed a biological property, as is commonly recognized (Searle, 2013), we should be able to apply Niko Tinbergen's four questions (Bateson and Laland, 2013):

1. What are the mechanisms (*causation*) of consciousness?
2. How is consciousness developed from birth (*ontogeny*)?
3. What is the survival value (*adaptivity*) of consciousness?
4. What is the evolutionary history (*phylogeny*) of consciousness?

The first and second questions are studied at the level of the organism at hand. However, when it comes to consciousness, these questions are hampered directly by the unresolved mind-body problem. Indeed, despite immense scientific, philosophical, and public interest in these questions, we still do not know how neural machinery can lead to consciousness. Progress has been made in identifying neural correlates of consciousness (NCCs) (Koch et al., 2016) but to date, this has not matured to a mechanistic explanation of consciousness (Chalmers, 2013). Moreover, due to the limited ability in measuring consciousness in animals, attempts to answer questions 1 and 2 have been mostly researched in humans. To date, there is no agreed upon knowledge on the relationship between behavior and consciousness or the brain and consciousness (Van Gulick, 2018). Therefore, whether behavioral observations or physiological results can teach us which and how animals are conscious is questionable (Dawkins, 2017; Gutfreund, 2017). However, hope may come by taking the evolutionary route to understanding consciousness, i.e., answering Tinbergen's questions 3 and 4. Having an evolutionary theory of consciousness, we could predict when it arises in evolution and which animals should have it.

The field of ethology established that animal behaviors, including learned behaviors, are shaped by evolution through natural selection (Burkhardt, 2005). Behavior directly affects the fitness of the animal and thus natural selection is concern with what the animal^1^ is doing and not what the animal is feeling. For consciousness to evolve in biological evolution it must have an adaptive value at the behavioral (observable) level. The major question, the answer to which is necessary for any evolutionary theory of consciousness, is what this adaptive value is. Two different approaches in coping with the question can be found in the literature. One is that consciousness has a function through which it enhances fitness (Seth, 2009). The other is that consciousness itself has no function, however, it is a byproduct of other, observable (brain) properties that do have an evolutionary function (Robinson et al., 2015).

## The proposition that consciousness has a function

**Figure 1:**
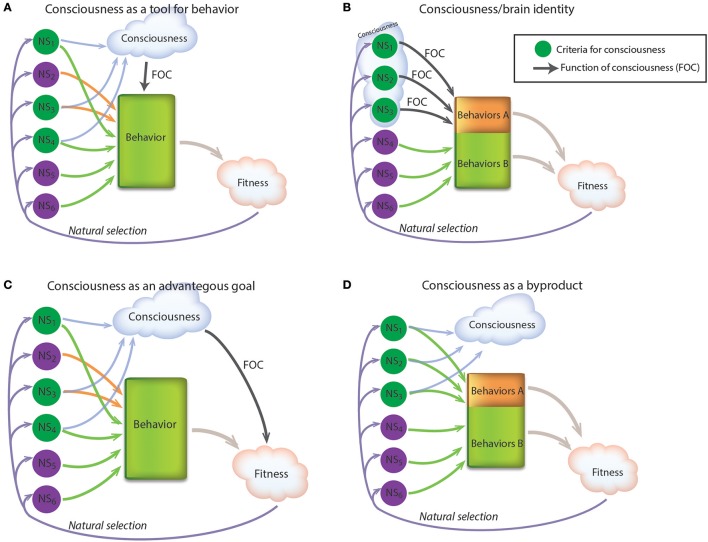
Attempts to fit consciousness into an evolutionary framework. NS, Neural State; FOC, Function of consciousness; Behaviors A, The group of behaviors which are associated or affected by consciousness; Behaviors B, The group of behaviors which are not associated with consciousness. (**A**) Consciousness as a tool for behavior. In this scheme consciousness arises from neural states and in turn affects behaviors. (**B**) Consciousness/brain identity. In this scheme certain neural states are conscious states. (**C**) Consciousness as an advantageous goal. In this scheme the property of being conscious contributes directly to fitness. (**D**) Consciousness as a by-product. In this scheme consciousness is a by-product without a function. It is maintained in evolution because of its association with advantageous behaviors.

A biological function can either be a function of a tool that allows a certain goal or a function of the goal itself. For example, the function of the bird wings is to enable flight, therefore, the wings are the tool for flight. Flight, on the other hand, is the goal of its underlying tools, and its function is to move the bird quickly and efficiently to food sources, to mates and away from danger. Is consciousness to an animal like wings are to a bird, i.e., a tool to enable an advantageous goal (Figure 1A)? If it is a tool, what is the goal that it enables? Some answers include: to create a unified and coherent representation of all incoming information (Crick and Koch, 1998; Merker, 2005); to enable the learning of sensory and cognitive representations (Grossberg, 1999); to make complex flexible decisions (Earl, 2014); and more. Consciousness is commonly considered a tool for flexible, context and memory-based cognitive behaviors that in turn are clearly adaptive (Seth, 2009). Difficulty with this notion is that cognitive behaviors are caused by the brain's neural circuits, without the necessity to introduce conscious states to the models. This gives rise to the paradox that if behavior is caused fully by unconscious neural circuits, how can it also be caused by feelings (Gutfreund, 2017)? One escape route around this paradox is to suggest an identity between consciousness and neuronal states (Loorits, 2014; Smart, 2017), that is, some neuronal states are conscious feelings; the two are the same, described at different levels (Figure 1B). The biological function of the neural state then becomes the function of the feeling (Searle, 2013). A problem with such an identity approach is that evolution operates at the level of the body and not at the level of the feelings. The only things that matter from an evolutionary point of view are the animal's actions, and the neural processes that choose and elicit the actions. Whether some of these neural processes can be described as subjective experiences at a higher psychological level is not relevant for the evolutionary story. Therefore, the implication of an identity hypothesis is that consciousness becomes detached from any evolutionary theory.

What if consciousness is a goal in itself? In this case, neurons organized in specific ways in specific brain structures are the wings to support consciousness, and the property of being conscious improves the fitness of the animal in which it is installed (Figure 1C), just like the properties of flying, swimming or chewing. But, in what ways do feelings and emotions improve fitness? An antelope escaping from a lion needs to run quickly and efficiently. Why, from an evolutionary point of view, does it also need to feel the terrible feeling of fear? This is a puzzle and evolutionary theory has no answers. Any attempt to answer this question without invoking an identity between conscious and neuronal states is hampered by the difficulty mentioned above, whereby a function must be realized at the behavioral level, but all biological behaviors are fully caused by their underlying neural behaviors rendering feelings, subjective experiences, intentions, etc. unnecessary for fitness.

## The proposition that consciousness doesn't have a function of its own

A different approach that bypasses the difficulties described above is to view consciousness as a byproduct of brain activity. In this case, consciousness doesn't affect behavior and has no function of its own. However, it has an adaptive value that stems from its association with a behavioral phenomenon, which in turn does have a function (Eccles, 1994; Robinson, 2007). The evolutionary theory of consciousness by Bronfman et al. (2016) is an example of implementation of such an approach. Bronfman et al. (2016) postulate that consciousness has no function of its own but is generated by the same brain features that are required for the cognitive property of unlimited associative learning (UAL). UAL then becomes a marker for consciousness, and tracking its evolution is synonymous with tracking the evolution of consciousness (Figure 1D). The pitfall of such an approach is that consciousness can be removed from the model without any influence on the flow of the model. The validity of the model as an evolutionary model of consciousness is critically dependent on a small set of features that are supposed to be necessary and jointly sufficient for minimal consciousness (criteria for consciousness) (Bronfman et al., 2016). Thus, the ability to track the evolutionary origins of consciousness rests on the question of whether we can identify the behavioral and physiological criteria that are necessary and sufficient for consciousness.

Donald Griffin laid down a road map for the ethological study of consciousness in his famous book, “The Question of Animal Awareness” (Griffin, 1981). A critical and first step to the scientific study of animal consciousness is to constitute what he called “a practical definition of consciousness.” Griffin was one of the firsts to present a list of criteria that he believed are sufficient for consciousness. Following Griffin, many added, modified, and used such criteria in numerous publications to conclude on animal consciousness as well as on the evolution of consciousness (Edelman and Seth, 2009; Butler, 2012; Feinberg and Mallatt, 2016). Common to all these criteria is that they are based on human consciousness, either through introspection or through the modern study of human NCCs. An example of the former is the “integration and binding of information” (Tononi et al., 2016); an example of the latter is the “neural modulation of thalamo-cortical loops” (Seth et al., 2005). Introspection carries the risk of obtaining false criteria because we are aware of our behaviors and not of the properties that constitute consciousness (Dennett, 2002). The fact that we consciously perceive an apple as a categorical whole does not exclude the possibility that in unconscious perception binding of information also occurs, nor does it exclude the possibility that conscious perception can happen without the binding of information. It simply reflects the fact that the integration of information for the control of adaptive behavior is a common property of brain function. On the other hand, using NCCs to illuminate brain criteria for consciousness in animals is impeded by the correlation-to-criterion fallacy. Correlation implies neither necessity nor sufficiency. Water in our environment is commonly correlated with liquids but we wouldn't say that all that is liquid is water. Finally, the features suggested as criteria for consciousness are complex and, in most cases, poorly-defined. Extrapolating such features as criteria for animal consciousness without knowledge about how they are linked to human consciousness is an oversimplification that most likely leads to premature conclusions about animal consciousness. Pelicans have a large wing span that enables fast flight. If we expand this criterion across species without knowledge about the physics of flight, we reach the false prediction that falcons are slow flyers and marabous are fast flyers.

## Conclusion

Consciousness is one of the last biological phenomena about which we do not have a solid idea as to how and when it appeared and evolved in evolution. The conclusion of the above discussion is that in order to identify the adaptive value of consciousness, the relationships between the brain, behavior, and consciousness must be understood. Thus, the question of how the mind emerged in evolution (the mind-evolution problem) is tightly linked with the question of how the mind emerges from the brain (the mind-body problem). It seems that the evolution of consciousness cannot be resolved without first solving the “hard problem” (Chalmers, 1995). Until then, I argue that strong claims about the evolution of consciousness based on the evolution of cognition are premature and unfalsifiable.

## Author contributions

The author confirms being the sole contributor of this work and approved it for publication.

### Conflict of interest statement

The author declares that the research was conducted in the absence of any commercial or financial relationships that could be construed as a potential conflict of interest.

## Footnotes

^1^By behavior, I mean the broadest sense of behavior, that is, every observable response of the body, including sensing and internal physiological responses.
